# Socio-demographic determinants and access to prenatal care in Italy

**DOI:** 10.1186/1472-6963-14-174

**Published:** 2014-04-15

**Authors:** Manuela Chiavarini, Donatella Lanari, Liliana Minelli, Luca Salmasi

**Affiliations:** 1 Department of Experimental Medicine, Public Health Section, University of Perugia, Perugia, Italy; 2 Department of Economics, Statistics and Finance, University of Perugia, Perugia, Italy

## Abstract

**Background:**

Many governments have made commitments to examine inequalities in healthcare access based on studies assessing the association between several socio-demographic factors and late initiation or fewer prenatal examinations. This study addressed the question of whether socio-demographic determinants were significant in explaining differences in prenatal care in one administrative region of Italy, Umbria.

**Methods:**

Data were obtained from the administrative source of the regional Standard Certificate of Live Births between 2005 and 2010, and were merged with Census data to include a socio-economic deprivation index. Standard and multilevel logistic regression models were used to analyze the magnitude of various individual-level maternal characteristics and socio-demographic indicators, such as nationality, employment status, education with respect to late access to the first examination, and low number of medical visits.

**Results:**

The study involved approximately 37,000 women. The heterogeneous effects of socio-demographic variables were documented on the prenatal care indicators analyzed. A multivariate model showed that women born outside Italy had a higher probability of making their first visit later than the 12th week of pregnancy and low numbers of prenatal medical visits; the estimated odds ratio for the analyzed indicators range from 2.25 to 3.05. Inadequate prenatal healthcare use was also observed in younger and pluriparous women and those with low education; in addition, having a job improved the use of services, possibly through transmission of information of negative consequences due to delayed or few prenatal visits. Interestingly, this study found a substantial reduction in the number of pregnant women who do not use prenatal healthcare services properly.

**Conclusions:**

The aim of this research is to provide more accurate knowledge about the inadequate use of prenatal healthcare in Italy. Results highlight the existence of differences in healthcare use during pregnancy, especially for women from less advantaged social classes (i.e., unemployed or poorly educated). Such inequalities should be examined in all areas of public policy and public services, to ensure equal opportunity for their use.

## Background

Prenatal healthcare (PNC) has the potential to reduce perinatal morbidity and mortality by identifying and reducing potential risks, treating medical conditions, and promoting healthier lifestyles. Prenatal care includes identification of medical conditions which require careful surveillance throughout pregnancy and which may be due to individual and/or contextual characteristics. The use of PNC has largely contributed to the decrease in perinatal and infant mortality rates in high-income countries over the last century, but access to PNC is still not equally practised by some groups of the population, even if prenatal care is universally and easily available. Promoting adequate access to PNC, especially among the most vulnerable groups of the population, may help to reduce differences in birth weight, infant mortality and morbidity rates, and serve as a guide for further improvements in the quality of care^a^. We follow [1] in defining adequate access to PNC as the fulfillment of at least four antenatal visits or the observance of the first visit before the 12th gestational week. Moreover, we will use the term “access” as “realized access” or “use” of PNC services.

The Italian Health Service (IHS) is based on the principle of universal coverage. The IHS is financed by general taxation, and has decentralized governance, ensuring that national guidelines and established targets are implemented throughout the country by way of the local administrations of nineteen regions and two autonomous provinces, which are responsible for managing assigned budgets, healthcare organization and local performance. Decentralized governance is then responsible for ensuring the delivery of PNC services by means of public and private accredited hospitals. The IHS provides universal coverage and free prenatal healthcare at point of delivery to all Italian and European Union nationals. Regardless of coverage, emergency treatment is available free of charge or at low-cost to anyone who requires it in Italy. In particular, the IHS offers 4 visits during pregnancy without any charge, and the program of these visits is the same for all women. During the first visit, which ideally is done before the 12th gestational week, women receive information about services offered and courses promoted by the IHS, and about screening tests. The other visits include discussion of screening tests or other results. Receiving fewer than 4 visits or making the first visit after the 12th gestational week will be considered hereafter as inadequate.

As in the study by [2], here we investigate the relationship between various determinants and the utilization of healthcare services. The framework of [3] on health behavior adopted in this work is useful because it incorporates the distinction between various individual determinants, e.g. the predisposition of the patient, the ability to secure service use, and possibly disease level. In particular, demographic, social and attitudinal variables are complemented if a person has a regular source of care, which reflects the potential accessibility of healthcare.

Previous studies have shown that young maternal age [4-6], low education [2,6-9], unemployed status [10,11], immigrant status [8,12,13], pluriparous status [5,8,14] and single marital status [6,15-17] are all barriers to early initiation of prenatal care and to an appropriate number of prenatal examinations.

To describe the influence of which determinants are related to late and/or inadequate PNC, together with individual-level maternal characteristics, this paper examines the role of predisposing, enabling, and pregnancy-related factors on adequate prenatal care use by including in the analysis differences in socio-economic conditions related to the mother’s place of residence in Umbria^b^[3].

Our contribution to the existing literature also lies in the estimation of the evolution over time of PNC use by exploiting the relatively long time-span covered by our data (2005–2010), from which we can provide insights on the advertising campaigns promoted by the Umbria region in 2005 regarding the benefits for mothers and newborn infants of following prenatal guidelines.

## Methods

### Data sources

Our study is based on data from the Standard Certificate of Live Birth (SCLB) forms of the Umbria region (Italy) between 2005 and 2010. This data source provides information on births for the entire population of the region. In Italy, state law requires certificates to be compiled for all births. To ensure uniform methodology applied to regional surveys and to obtain datasets containing comparable indicators, all regions are required to compile the same form, the “Standard Certificate of Live Birth” (SCLB). The midwife or obstetrician who attends the birth, or the doctor responsible for delivery in the operating theatre (in hospital), must complete the SCLB within 10 days of delivery, reporting epidemiological information regarding the mother’s state of health, socio-demographic characteristics, risk factors during the pregnancy, obstetric procedures, characteristics and method of delivery (e.g., normal delivery or caesarian section), and inclusion of any abnormal conditions or congenital anomalies of the infant, cause of mortality, information about use of prenatal care services, etc. (for further details, see Decree No. 349 of the Italian Ministry of Health, [18]). We used population data from the Umbria region, which merges data from each mother and her baby for a total of 37,000 records.

### Variables of interest and descriptive analyses

We followed the healthcare indicators recommended by [1] for monitoring and evaluating maternal and child health in the perinatal period and considered two binary indicators of access to prenatal care from the SCLB^c^[6]:

1. *Number of prenatal visits*: low number of prenatal medical visits^d^[14] (LPV) (below 4) and standard number of prenatal visits (SPV) (at least 4).

2. *Timing of first visit*: late first visit^e^[19] (LFV) (after more than 12 weeks) and regular timing of first visit (RFV) (fewer than 12 weeks)^f^[2].

As determinants, we considered a set of individual-level variables as follows: age, with four categories: ≤ 20, 20–29, 30–39, > 39 (reference category: age 20–29); nationality of mother, with three categories: Italian (ITA), European Union (EU-27), and rest of the world (Extra EU-27) (reference category: Italy); marital status, with two categories: married, unmarried (reference category: married). Education was measured as self-reported level of education, according to the International Standard Classification of Education (ISCED): low (not more than 8 years of education), medium (from 9 to 13 years), and high (more than 13 years); the latter was used as the reference category. Employment status was classified into five categories: self-employed or white-collar workers, blue-collar workers, unemployed, looking for a first job, and students or housewives (reference category: self-employed or white collar). We also examined the impact of pregnancy factors on preterm birth by including women with previous pregnancies (pluriparous women, category 1+; absence of previous pregnancies, 0. The latter is taken as reference category).

We were interested in assessing whether socio-economic disadvantages (*sed*) are linked with PNC use in Umbria. We therefore calculated a deprivation index for the 92 municipalities of the region on the basis of census data for the year 2001, provided by the Italian Institute of Statistics. Five variables^g^[11], suitably standardized^h^[15], were used to construct the index. The final index was obtained as the sum of the standardized scores and then categorized on the basis of the quartiles of the observed score distribution (*sed*_*1*_, *sed*_*2*_, *sed*_*3*_, *sed*_*4*_). We used the first quartile (*sed*_*1*_) as the reference category; the other categories indicate increasing levels of socio-economic deprivation.

### Statistical methods

In order to analyze the relationship between PNC (Y) use and individual and contextual characteristics (X), we first used a basic logistic regression model. This model was then augmented by including municipal-level fixed effects (i.e., FE-Logit), which accounted for the influence of territorial differences. For the sake of simplicity, we omitted time effects and wrote the model as follows:

(1) $$\ln\left[\frac{P\left(Y_{i}|X_{i}\right)}{1-P\left(Y_{i}|X_{i}\right)}\right]=\beta_{0}+\sum_{k=1}^{K_{1}}\beta_{k}X_{\mathrm{ki}}+\sum_{k=K_{1}+1}^{K_{2}}\beta_{k}M_{k}+\epsilon_{i}.$$

where covariate vector *X*_*ki*_ contains the variables at the individual level already described in the previous subsection, and five time dummies. With this specification, the set of dummy variables *M*_*k*_ mimics the influence of the unobservable characteristics of the mother’s place of residence and *ϵ*_*i*_ is the residual term.

Since we were also interested in characterizing socio-economic inequalities on PNC services - which may depend on where a woman lives - we proposed a multilevel logistic model. Equation 2 defines a logistic regression with two levels of aggregation, individual *i* and municipality *j*, written in log-odds form as:

(2) $$\ln\left[\frac{P\left(Y_{\mathrm{ij}}|X_{\mathrm{ij}}\right)}{1-P\left(Y_{\mathrm{ij}}|X_{\mathrm{ij}}\right)}\right]=\beta_{0}+\sum_{k=1}^{K}\beta_{\mathrm{kij}}X_{\mathrm{kij}}+u_{j},$$

with

(3) $$u_{j}\approx N\left(0,\sigma_{u}\right)$$

where *u*_*j*_ is the error term at the municipal level and *σ*_*u*_ is the between-group variance or residual variance across municipal areas, which allowed us to estimate the model under random effects (RE-Logit)^i^[20].

This framework had the advantage of potentially identifying determinants of PNC inequalities through the inclusion of municipal-level variables, which in our analysis are represented by the deprivation index. The aim of this strategy was to test whether, after including municipal-level indicators, we could reduce the unexplained variance of *Y* across geographical areas of Umbria (RE_M_-Logit specification). In addition, each model includes time dummies. According to variations in the odds ratio (OR), we ascertained whether PNC use changed over time and examined the degree of success of recent regional efforts to ensure that pregnant women were directly informed by their doctors or indirectly by publicity campaigns about the health advantages of following PNC recommendations.

## Results

The study population from which our sample was drawn collected information on around 550,000 births from 549 hospitals in Italy. The percentage of women making the first visit later than the 12th gestational week in the population is 2.9%, and increases to about 15% for foreign-born, poorly educated and young women [21]. In this paper we use a subsample obtained from the Umbria region that collects information on approximately 8,000 deliveries each year.

Descriptive statistics for the two PNC outcomes of interest from our sample, conditional on covariates, are listed in Table 1. We observe that women making a lower number of visits tend to be younger and less educated with respect to women making a recommended number of prenatal visits. The same result holds true for women born outside of Europe, 16.94% of whom had fewer than 4 visits, compared with 5.29% for women of Italian nationality. Higher percentages of employed mothers follow recommendations and have four or more antenatal examinations. Differences in the use of PNC between married and unmarried women seems to be irrelevant, whereas pluriparous women seem to make fewer antenatal visits with respect to their reference category. Similar patterns are observed in relation to the timing of the first examination, suggesting a large positive correlation between these indicators. Living in more deprived areas does not seem to have any particular influence on PNC services. Results from the descriptive analysis will be formally tested with multivariate logistic regression models.

**Table 1:** Descriptive statistics

**Variable**	**Category**	**Number of visits**					**First visit**				
		**< 4**		**≥ 4**		**Total**	**≥ 12 weeks**		**< 12 weeks**		**Total**
		**n**	**%**	**n**	**%**	**n**	**n**	**%**	**n**	**%**	**n**
Maternal age	≤ 20	206	19.64	843	80.36	1049	149	14.23	898	85.77	1047
	20 - 29	1418	10.73	11802	89.27	13220	1147	8.71	12022	91.29	13169
	30 - 39	1415	5.93	22438	94.07	23853	1239	5.21	22564	94.79	23803
	> 39	201	7.36	2529	92.64	2730	154	5.65	2570	94.35	2724
Maternal nationality	ITA	1630	5.29	29182	94.71	30812	1341	4.36	29402	95.64	30743
	EU-27	473	16.94	2319	83.06	2792	371	13.36	2406	86.64	2777
	Extra EU-27	1170	17.36	5571	82.64	6741	1069	15.93	5643	84.07	6712
Maternal education	High	515	4.71	10427	95.29	10942	491	4.5	10431	95.5	10922
	Medium	1528	6.91	20590	93.09	22118	1175	5.32	20891	94.68	22066
	Low	1376	13.15	9091	86.85	10467	1159	11.1	9279	88.9	10438
Maternal employment	White-collar	288	5.12	5341	94.88	5629	247	4.4	5373	95.6	5620
	Blue-collar	1128	5.04	21238	94.96	22366	925	4.14	21396	95.86	22321
	Unemployed	523	12.4	3695	87.6	4218	423	10.06	3780	89.94	4203
	Student	75	11.19	595	88.81	670	58	8.68	610	91.32	668
	Housewife	1402	13.24	9190	86.76	10592	1165	11.03	9397	88.97	10562
Marital status	Married	2528	7.44	31441	92.56	33969	2189	6.46	31707	93.54	33896
	Unmarried	900	9.28	8802	90.72	9702	639	6.61	9026	93.39	9665
Parity	0	1746	6.67	24430	93.33	26176	1386	5.31	24712	94.69	26098
	1+	1693	9.61	15927	90.39	17620	1464	8.33	16114	91.67	17578
Deprivation index	*sed1*	959	8.66	10115	91.34	11074	738	6.68	10317	93.32	11055
	*sed2*	870	7.3	11046	92.7	11916	969	8.16	10910	91.84	11879
	*sed3*	970	7.6	11799	92.4	12769	613	4.82	12115	95.18	12728
	*sed4*	642	7.98	7407	92.02	8049	530	6.6	7495	93.4	8025
Year	2005	659	9.22	6492	90.78	7151	604	8.48	6520	91.52	7124
	2006	571	7.92	6638	92.08	7209	564	7.84	6626	92.16	7190
	2007	560	7.9	6531	92.1	7091	474	6.7	6598	93.3	7072
	2008	601	7.85	7054	92.15	7655	411	5.39	7216	94.61	7627
	2009	531	7.2	6842	92.8	7373	432	5.87	6929	94.13	7361
	2010	519	7.08	6810	92.92	7329	365	4.99	6948	95.01	7313
Total		2850	6.52	40837	93.48	43687	3441	7.85	40367	92.15	43808

Note: The socio-economic deprivation index is built as already described in the Methods section.

Table 1 also shows that there is an increasing trend in PNC use for both indicators. Overall, we see that 93.48% of pregnant women make more than four prenatal visits and that 92% make their first visit before the 12th gestational week. However, for the most disadvantaged groups of the population (i.e., poorly educated, unemployed, foreign-born and unmarried) such percentages decrease to a range from 80% to 90% (see Table 1, columns 6 and 11).

Table 2 lists the estimated odds ratio when the dependent variable is the number of prenatal examinations. The estimates under the FE-Logit model are shown in the second column; column 3 lists those obtained under the random effects specification (RE-Logit), extended in column 4 to include the deprivation index at the municipal level (RE_M_-Logit). Since we are particularly interested in testing whether deprivation is correlated with the number of prenatal visits, conditional on individual characteristics, we discuss here only the estimates of the multi-level model, based on evidence that the difference between the magnitudes of the coefficients of socio-demographic variables is statistically negligible across the other specifications (see Table 2).

**Table 2:** Estimates of socio-demographics for access to prenatal care

**Variables**	**Logit**	**FE-logit**	**RE-Logit**	**RE**_**M**_**-Logit**	**CI (95%)**	
Age ≤ 20	1.40***	1.38***	1.38***	1.38***	1.13	1.7
	(0.141)	(0.144)	(0.143)	(0.143)		
Age 30 – 39	0.86***	0.84***	0.84***	0.84***	0.76	0.93
	(0.042)	(0.043)	(0.042)	(0.042)		
Age > 39	0.88	0.84*	0.84*	0.84*	0.7	1.03
	(0.087)	(0.084)	(0.083)	(0.083)		
Nationality: EU-27	2.72***	2.58***	2.59***	2.59***	2.25	2.98
	(0.183)	(0.186)	(0.186)	(0.186)		
Nationality: Extra EU-27	2.94***	2.94***	2.92***	2.92***	2.63	3.26
	(0.150)	(0.162)	(0.161)	(0.161)		
Education: Medium	0.94	0.99	0.99	0.99	0.88	1.12
	(0.057)	(0.061)	(0.061)	(0.061)		
Education: Low	1.26***	1.37***	1.36***	1.36***	1.2	1.55
	(0.082)	(0.091)	(0.091)	(0.091)		
Occupation: Blue collar	0.86*	0.87*	0.87*	0.88*	0.75	1.02
	(0.067)	(0.069)	(0.069)	(0.069)		
Occupation: Unemployed	1.25**	1.60***	1.56***	1.56***	1.29	1.88
	(0.115)	(0.154)	(0.149)	(0.149)		
Occupation: Student	1.51**	1.34*	1.35*	1.35*	0.97	1.87
	(0.254)	(0.225)	(0.225)	(0.225)		
Occupation: Housewife	1.36***	1.33***	1.34***	1.34***	1.14	1.58
	(0.110)	(0.112)	(0.112)	(0.113)		
Marital status: Unmarried	1.17***	1.15**	1.15***	1.15***	1.04	1.28
	(0.060)	(0.061)	(0.061)	(0.061)		
Parity: 1+	1.57***	1.59***	1.58***	1.58***	1.44	1.73
	(0.071)	(0.073)	(0.072)	(0.072)		
Deprivation index: *sed*_*2*_				1.35	0.6	3.06
				(0.564)		
Deprivation index: *sed*_*3*_				1.29	0.88	1.9
				(0.255)		
Deprivation index: *sed*_*4*_				1.14	0.7	1.84
				(0.279)		
Year: 2006	1	0.97	0.98	0.98	0.85	1.11
	(0.066)	(0.065)	(0.066)	(0.066)		
Year: 2007	0.83***	0.78***	0.78***	0.78***	0.68	0.9
	(0.058)	(0.055)	(0.056)	(0.056)		
Year: 2008	0.62***	0.60***	0.60***	0.60***	0.52	0.7
	(0.045)	(0.044)	(0.044)	(0.044)		
Year: 2009	0.74***	0.71***	0.72***	0.72***	0.63	0.83
	(0.052)	(0.051)	(0.051)	(0.051)		
Year: 2010	0.58***	0.55***	0.55***	0.55***	0.48	0.64
	(0.043)	(0.041)	(0.042)	(0.042)		
Constant	0.05***	0.01***	0.04***	0.03***	0.03	0.05
	(0.005)	(0.010)	(0.005)	(0.006)		
σ_u_			0.68	0.68	0.54	0.84
			(0.077)	(0.076)		
Ρ			0.12	0.12	0.08	0.18
			(0.024)	(0.024)		
LR test for ρ = 0			478.88***	393.75***		
Observations	36,993	36,717	36,993	36,993		
Adj. R-squared	0.08	0.12	.	.		
Number of municipalities			91	91		

Notes: The confidence interval (CI) at 95% significance level refers to the Re_M_-Logit model (see text). Standard errors in brackets. Significant levels as follows: p-value *** ≤ 0.01, ** ≤ 0.05, * ≤ 0.1.

Outcome of interest: odds ratios between fewer than four prenatal visits (LPV) versus four or more prenatal visits (NPV).

Second-level variance component σ_u_ is 0.68 (s.e. = 0.076), and intra-class correlation coefficient ρ is 0.12 (s.e. = 0.024). This means that 12% of the variability in differences in healthcare use is not explained by the individual variables. More importantly, the coefficients associated with the municipal-level deprivation index are not significant, and the estimates of the other individual-level maternal characteristics do not change when it is included.

Many of the individual socio-demographic variables clearly have significant links with LPV. From the results shown in Table 2, women in the younger classes are more likely to be in the LPV category. The age class ≤ 20 has higher odds (38%) of being in the LPV category than the reference age class (20–29). A significant relationship between the mother’s education and the number of prenatal visits can also be observed. Women with fewer than 8 years of education have estimated LPV odds that are 36% higher than those of highly educated women.

Another important risk factor for LPV is found to be associated with the mother’s occupation. Women classified as unemployed or looking for their first job [OR = 1.56, (95% CI = 1.29-1.88)], students [OR = 1.35, (95% CI = 0.97-1.87)] or housewives [OR = 1.34, (95% CI = 1.14-1.58)] have higher odds of making LPV with respect to the reference category (self-employed or white-collar workers). In contrast, women within the blue-collar category tend to follow the guidelines of making at least four visits annually [OR = 0.88, (95% CI = 0.75-1.02)]^j^[22], like those in the reference category.

Women born outside Italy, from both EU-27 and outside it, have odds of LPV three times higher than those of Italian women [OR = 2.59 and 2.92, for women from EU-27 and from the rest of the World, respectively]. The odds ratio for unmarried women is significant and slightly higher than one [OR = 1.15, (95% CI = 1.04-1.28)]. In addition, pluriparous women have higher odds of being in the LPV category [parity, OR = 1.58, (95% CI = 1.44-1.73)].

Lastly, the estimated odds ratio from time dummies indicates a substantial increase across years in the use of PNC services. With respect to 2005, LPV odds in 2009 and 2010 are 28% [OR = 0.72, (95% CI = 0.63-0.83)] and 45% [OR = 0.55, (95% CI = 0.48-0.64)] lower, respectively.

Table 3 lists the estimates of the relationship between socio-demographic variables and the use of PNC when LFV is used as the outcome of interest. First, as in the previous case, younger [OR = 1.65, (95% CI = 1.39-1.97)] and unmarried women [OR = 1.44, (95% CI = 1.32-1.58)] have higher odds of making their first visit later in pregnancy than recommended. Second, women with educational level between 9 and 13 years (i.e., medium), with respect to highly educated ones [OR = 1.15, (95% CI = 1.03-1.29)] have higher odds of making a late first visit. Third, although the intra-class coefficient ρ is significantly different from zero, according to the likelihood ratio test, σ_u_ falls to 0.26 (s.e. = 0.042) and intra-class correlation coefficient ρ falls to 0.02 (s.e. = 0.006) with respect to the same parameters listed in Table 2. This means that for the LFV category, a larger percentage of variability is explained by individual variables and time dummies. Fourth, the estimated coefficients associated with time dummies indicate that the odds of late access decreased by about 30% between 2005 and 2010. All the other estimated odds ratios are very similar to those already described for the previous indicator and, for the sake of simplicity, will not be commented on here.

**Table 3:** Estimates of socio-demographics for access to prenatal care

**Variables**	**Logit**	**FE-Logit**	**RE-Logit**	**RE**_**M**_**-Logit**	**CI (95%)**	
Age ≤ 20	1.65***	1.64***	1.65***	1.65***	1.39	1.97
	(0.149)	(0.149)	(0.149)	(0.149)		
Age 30 – 39	0.79***	0.78***	0.79***	0.79***	0.72	0.86
	(0.036)	(0.036)	(0.036)	(0.036)		
Age > 39	0.96	0.97	0.97	0.97	0.82	1.15
	(0.084)	(0.084)	(0.084)	(0.084)		
Nationality: EU-27	2.67***	2.70***	2.69***	2.69***	2.38	3.05
	(0.169)	(0.174)	(0.172)	(0.172)		
Nationality: Extra EU-27	2.49***	2.56***	2.55***	2.55***	2.31	2.82
	(0.127)	(0.131)	(0.130)	(0.130)		
Education: Medium	1.17***	1.16**	1.15**	1.15**	1.03	1.29
	(0.067)	(0.068)	(0.067)	(0.067)		
Education: Low	1.49***	1.46***	1.46***	1.45***	1.28	1.65
	(0.094)	(0.094)	(0.093)	(0.093)		
Occupation: Blue collar	0.88*	0.88*	0.88*	0.88*	0.76	1.02
	(0.064)	(0.064)	(0.065)	(0.065)		
Occupation: Unemployed	1.33***	1.41***	1.39***	1.39***	1.17	1.65
	(0.115)	(0.126)	(0.123)	(0.123)		
Occupation: Student	1.54***	1.55***	1.55***	1.55***	1.15	2.09
	(0.233)	(0.235)	(0.235)	(0.235)		
Occupation: Housewife	1.45***	1.39***	1.42***	1.42***	1.22	1.65
	(0.112)	(0.110)	(0.111)	(0.111)		
Marital status: Unmarried	1.41***	1.45***	1.44***	1.44***	1.32	1.58
	(0.066)	(0.068)	(0.068)	(0.068)		
Parity: 1+	1.53***	1.52***	1.53***	1.53***	1.41	1.66
	(0.064)	(0.064)	(0.064)	(0.064)		
Deprivation index: *sed2*				0.89	0.64	1.25
				(0.154)		
Deprivation index: *sed3*				0.92	0.76	1.12
				(0.091)		
Deprivation index: *sed4*				1.05	0.83	1.34
				(0.130)		
Year: 2006	0.87**	0.89*	0.88**	0.88**	0.78	1
	(0.057)	(0.058)	(0.057)	(0.057)		
Year: 2007	0.86**	0.86**	0.86**	0.86**	0.75	0.98
	(0.057)	(0.057)	(0.057)	(0.057)		
Year: 2008	0.84***	0.83***	0.83***	0.83***	0.73	0.94
	(0.054)	(0.054)	(0.054)	(0.054)		
Year: 2009	0.73***	0.73***	0.73***	0.73***	0.64	0.83
	(0.049)	(0.049)	(0.049)	(0.049)		
Year: 2010	0.70***	0.69***	0.69***	0.69***	0.6	0.78
	(0.047)	(0.046)	(0.046)	(0.046)		
Constant	0.05***	0.03***	0.05***	0.05***	0.04	0.06
	(0.005)	(0.014)	(0.005)	(0.006)		
σ_u_			0.271	0.26	0.19	0.36
			(0.042)	(0.042)		
Ρ			0.021	0.02	0.01	0.04
			(0.007)	(0.006)		
LR test for ρ = 0			83.26***	66.02***		
Observations	37,088	37,024	37,088	37,088		
Adj. R-squared	0.08	0.09	.	.		
Number of municipalities			91	91		

Notes: The confidence interval (CI) at 95% significance level refers to the Re_M_-Logit model (see text). Standard errors in brackets. Significant levels as follows: p-value *** ≤ 0.01, ** ≤ 0.05, * ≤ 0.1.

Outcome of interest: odds ratios between being late in first visit (LFV) versus regular timing of first visit (RFV).

## Discussion

This study provides evidence that the use of PNC services depends on individual socio-demographic and contextual characteristics. Our results highlight how, although prenatal healthcare services in Umbria are cost-free and available to all women, access may be prevented for some particularly vulnerable groups of the population. Given that inadequate use of PNC has a negative effect on birth outcomes (e.g., birth-weight or preterm deliveries) in Umbria, as shown by [22], it becomes relevant to understand what characteristics determine inadequate access.

In this study, in accordance with [1], we defined inadequate access as either making a low number of visits (i.e., fewer than four prenatal visits) or a late first visit (i.e., first visit after the 12th gestational week) and used multi-level logistic regressions to test whether variables with different levels of aggregation (individual and municipal) were significant in explaining differences in PNC use.

When we tested whether municipal-level deprivation affected the probability of PNC use, we found no significant effects. Our results are in accordance with those presented in the review of the literature proposed recently by [16,23]. However, we cannot exclude that an influence exists, at least at the individual level. In fact, [24] found that the perception of care differs according to the level of ‘engagement’^k^[17] and showed how lower levels of this perception are evident in most of the ‘least deprived’ groups and in almost none of the ‘most deprived’ groups. However, in his study this measure also becomes statistically less important when aggregate indicators are used.

We also analyzed the effect of individual-level variables on PNC use. Our results show how young, poorly educated, unmarried, pluriparous and unemployed women have higher probabilities of making inadequate use of PNC services. These results were also confirmed by previous studies [7,17,25], which showed that young age and pluriparous women were at significantly higher risk of inadequate PNC use. Moreover, [26] showed that young age was also associated with higher rates of low birth weight and preterm deliveries. Poorly educated women had a higher propensity to make LPV and LFV; however, the magnitude of our estimated odds ratio is slightly larger than that obtained from other countries: see [27]. Our estimates also indicate a positive relation between marital status and adequate use of PNC, a result confirmed also by [4,28]. Disrupted family situations, such as single parenting or a poor relationship with the baby’s father, significantly affect the use of PNC services [29,30]. The association of pluriparous pregnant women with low use of PNC has been reported in studies from Turkey, Indonesia and Brazil [19,20,31]. Pluriparous women may tend to rely on their previous experiences and feel more confident during the new pregnancy. In addition, our results show that mothers of non-Italian (foreign) nationality are more likely to have LPV and LFV. This result may be a consequence of poor language proficiency which, as already highlighted by [8], is a huge barrier to PNC use. Unemployment is found to be associated with higher probabilities of LPV and LFV. In fact, employed mothers may use PNC services more frequently because information about pregnancy risks is widely available in the workplace. One fact which emerges from this study is that the use of prenatal healthcare services has steadily increased over the last years in Umbria. Although we cannot attribute a causal effect, our results indicate that information campaigns about the benefits for women and newborn infants of following prenatal guidelines, promoted by the Umbria region in 2005, may have had a positive influence on PNC use.

## Conclusions

This study showed how the use of PNC services is associated with individual characteristics and less influenced by contextual variables related to the level of deprivation of the mother’s area of residence. We also showed that in the past years the use of PNC services has strongly increased in Umbria. This result is relevant from a policy perspective, since promotion of adequate PNC use can contribute to reducing health inequalities for newborn infants.

One limitation of our work is that, although our sample utilizes administrative data and includes all pregnant women in Umbria, it lacks information about the infants’ fathers, who may be responsible for other aspects related to deprivation. Moreover, given that a relevant percentage of women are already receiving adequate care, according to the definition of inadequate use of PNC adopted in this study, it would be worthwhile also to incorporate information about content and quality of care received. Future research on this topic, using SLBC data, should include information about the infants’ fathers and further enhance the definition of inadequate care by including information about quality and quantity of care received and providing estimates of the influence of inadequate access on preterm deliveries or low birth weight.

## Endnotes

^a^This perspective is not completely shared in the literature. For example, [32] showed that in China, since the early 1980s, utilization of maternal healthcare services has grown and antenatal and maternal health indicators have improved, whereas the meta-analysis by [33] found that reducing the number of prenatal examinations did not lead to increased adverse outcomes for the infant.

^b^For a comprehensive review, see [34].

^c^The recommended indicators of perinatal health also include those related to the management of sub-fertility and the care of preterm infants, describing variations in the use and success of these medical technologies in chronological order from pre-conception to postpartum care. For discussions, see [35,36].

^d^As reported by [37,38]: “there is still no consensus about the optimal number of prenatal visits”. The choice of the threshold varies among medical studies, our paper refers to the “WHO Antenatal Care Randomized Trial: Manual for the implementation of the New Model”, used to justify the choice of the threshold of four recommended prenatal visits.

^e^This indicator is also recommended by [39].

^f^We decided to analyze these indicators separately because we were interested in assessing if there were significant differences, from a statistical perspective, about the effect of socio-demographics on PNC access use, which a joint analysis would have not been able to do.

^g^The variables included in the deprivation index are: % of individuals who did not complete compulsory education, % of unemployed individuals or looking for their first job, number of individuals per dwelling, % of rented accommodation, % of single parents living with at least one child.

^h^Each component of the index was standardized as follows: z_i_ = (x_i_-μ_xi_)/σ_xi_, where z_i_, with i = 1,…,5 is the standardized i-th component of the deprivation index, x_i_ is the raw i-th index, and μ_xi_ and σ_xi_ are the means and standard deviations of raw index i.

^i^See [40].

^j^A test between blue-collars and self-employed or white-collars coefficients did not reject the null hypothesis of equality.

^k^Engagement is defined as personalization and active involvement in care, power and relationships, as well as healthcare literacy.

## Competing interests

The authors declare that they have no competing interests.

## Authors’ contributions

All the authors contributed to designing the structure of the study. MC and LM contributed to conceptualizing the ideas of this study. MC defined the background of the study. LS performed the statistical analysis, described results and reviewed the manuscript. DL interpreted and discussed results and reviewed the manuscript. LM interpreted results and also obtained funding. All authors read and approved the final manuscript.

## Pre-publication history

The pre-publication history for this paper can be accessed here:

http://www.biomedcentral.com/1472-6963/14/174/prepub
